# Editorial: Sustainable production of microorganism biomass for industrial fermentation

**DOI:** 10.3389/fmicb.2026.1783898

**Published:** 2026-02-04

**Authors:** Emma E. Tymczyszyn, María de los Ángeles Pozo-Bayón, Barbara Bravo-Ferrada, Natalia S. Brizuela

**Affiliations:** 1 Department of Science and Technology, National University of Quilmes, Bernal, Argentina; 2 Instituto de Investigacion en Ciencias de la Alimentacion, Madrid, Spain

**Keywords:** by-product, fermentation, food, microorganism, sustainable

Microbial fermentation has long been recognized as a fundamental pillar of industrial biotechnology, particularly, within the food and beverage sector. The Research Topic “*Sustainable Production of Microorganism Biomass for Industrial Fermentation*,” addresses the multifaceted role of microorganisms such as lactic acid bacteria (LAB), yeasts, and molds in the production of diverse bioproducts, including biofuels, enzymes, functional metabolites, and starter cultures. This Research Topic brings together recent advances focused on innovative strategies for microbial biomass production, process optimization, and sustainable fermentation technologies applicable at the industrial scale.

Several contributions within this Research Topic focus on the use of microorganismsin the fermentation of food and beverages. LAB and yeasts play essential roles in the transformation of grains, fruits, and dairy by-products, leading not only to the production of lactic acid or ethanol but also to novel fermented products with enhanced technological, functional, and nutritional properties. The studies presented explore innovative fermentation approaches aimed at improving product quality while reducing production costs.

Sustainability emerges as a central theme throughout this Research Topic, particularly through the valorization of agro-industrial by-products and waste streams. Several contributions demonstrate how substrates such as whey permeate, apple pomace, barley bran, fermented feedstock, and plant biomass residues can be transformed into valuable microbial biomass, biofuels, enzymes, prebiotics, or bioactive compounds. These approaches help reduce the the environmental footprint of food and agricultural industries while generating high-value products aligned with circular economy principles.

Preservation and stabilization of microbial biomass represent another critical aspect addressed in this Research Topic. The optimization of fluidized bed drying for *Lactiplantibacillus plantarum* grown in sustainable culture media exemplifies how alternative drying technologies can offer cost-effective and scalable solutions to conventional freeze-drying, while maintaining long-term viability and technological functionality for applications such as malolactic fermentation in winemaking.

Several studies also highlight advances in microbial engineering and process design aimed at improving industrial biomanufacturing. The development of antibiotic-free plasmid maintenance systems in industrially relevant *Escherichia coli* strains addresses both economic and regulatory challenges associated with antibiotic use, offering a robust and scalable strategy compatible with large-scale protein production. Such approaches are particularly relevant in the context of growing concerns about antimicrobial resistance and environmental contamination.

The biotechnological potential of non-conventional microorganisms is further explored in this Research Topic. The physiological characterization of *Kluyveromyces marxianus* strains demonstrates their suitability as microbial platforms for the conversion of lactose-rich whey permeate into bioethanol, supporting the sustainable production of biofuels from dairy industry by-products. Other contributions investigate complex microbial consortia and synergistic fermentations to improve feed quality, reduce greenhouse gas emissions, and enhance fermentation efficiency in animal production systems.

Beyond food, feed, and bioenergy applications, this Research Topic also showcases the role of microbial fermentation in the production of bioactive compounds. The use of endophytic fungi to bioconvert plant biomass residues into metabolites with anticancer, anti-inflammatory, and hemoprotective activities highlights the expanding scope of industrial fermentation toward pharmaceutical and nutraceutical applications.

Finally, the application of advanced analytical tools, including metagenomics, microbiome profiling, and metabolomics, provides deeper insight into microbial community dynamics and metabolite formation during fermentation. These approaches enable a more precise understanding of structure–function relationships in complex fermentations, supporting process optimization, quality control, and the rational design of starter cultures for industrial use.

The contributions collected herein reflect both traditional and emerging fermentation technologies and illustrate how microbial systems can be harnessed to develop efficient, environmentally responsible, and economically viable bioprocesses across multiple industrial sectors.

